# Response format changes the reading the mind in the eyes test performance of autistic and non-autistic adults

**DOI:** 10.1177/13623613231167226

**Published:** 2023-05-03

**Authors:** Alliyza Lim, Neil Brewer, Denise Aistrope, Robyn L Young

**Affiliations:** Flinders University, Australia

**Keywords:** autism, reading the mind in the eyes, response format, theory of mind

## Abstract

The Reading the Mind in the Eyes Test (RMET) is a purported theory of mind measure and one that reliably differentiates autistic and non-autistic individuals. However, concerns have been raised about the validity of the measure, with some researchers suggesting that the multiple-choice format of the RMET makes it susceptible to the undue influence of compensatory strategies and verbal ability. We compared the performance of autistic (*N* = 70) and non-autistic (*N* = 71) adults on the 10-item multiple-choice RMET to that of a free-report version of the RMET. Both the autistic and non-autistic groups performed much better on the multiple-choice than the free-report RMET, suggesting that the multiple-choice format enables the use of additional strategies. Although verbal IQ was correlated with both multiple-choice and free-report RMET performance, controlling for verbal IQ did not undermine the ability of either version to discriminate autistic and non-autistic participants. Both RMET formats also demonstrated convergent validity with a well-validated adult measure of theory of mind. The multiple-choice RMET is, however, much simpler to administer and score.

**Lay abstract:**

Recognizing and understanding the perspectives of others—also called theory of mind—is important for effective communication. Studies have found that some autistic individuals have greater difficulty with theory of mind compared to non-autistic individuals. One purported theory of mind measure is the Reading the Mind in the Eyes Test (RMET). This test presents participants with photographs of pairs of eyes and asks them to identify the emotion displayed by each pair of eyes from four choices. Some researchers have argued that the multiple-choice format of the RMET may not be an accurate measure of theory of mind, as participants could simply be guessing or using a process of elimination to select the correct answer. Participants may also be disadvantaged if they are not familiar with the specific emotion words used in the multiple-choice answers. We examined whether a free-report (open-ended) format RMET would be a more valid measure of theory of mind than the multiple-choice RMET. Autistic and non-autistic adults performed better on the multiple-choice RMET than the free-report RMET. However, both versions successfully differentiated autistic and non-autistic adults, irrespective of their level of verbal ability. Performance on both versions was also correlated with another well-validated adult measure of theory of mind. Thus, the RMET’s multiple-choice format does not, of itself, appear to underpin its ability to differentiate autistic and non-autistic adults.

Recognizing and understanding the perspectives of others—or theory of mind (Golan et al., 2006)—is important for effective communication. One widely used measure of theory of mind is the Reading the Mind in the Eyes Test (RMET; Baron-Cohen, Wheelwright, Hill, et al., 2001), which presents participants with photographs of pairs of eyes and asks them to identify the emotion displayed by each pair of eyes from four response options. Concerns have been raised about the validity of the measure (Gernsbacher & Yergeau, 2019), with some researchers suggesting that it does not actually measure theory of mind, but rather, emotion recognition (Oakley et al., 2016), intelligence (Rosso & Riolfo, 2020), and vocabulary (Olderbak et al., 2015).

Another concern raised about the RMET is that the multiple-choice response format provides respondents with contextual information that influences their test performance (Betz et al., 2019; Cassels & Birch, 2014). Cassels and Birch (2014) explored these concerns by comparing non-autistic children’s performance on the multiple-choice RMET with a free-report version, arguing that free-report performance would be less (a) vulnerable to the influence of deductive reasoning or process of elimination strategies, and (b) less dependent on receptive vocabulary. They found that children (aged 4–12 years) scored lower on the free-report than the multiple-choice RMET. Moreover, unlike the free-report RMET, the multiple-choice RMET was strongly associated with verbal ability. They proposed that the free-report RMET may therefore be advantageous when trying to identify emotion recognition deficits and when working with populations with limited verbal ability.

Betz et al. (2019) also found that non-autistic adults (aged 18–63 years) scored higher on the multiple-choice than the free-report RMET. They argued that the former’s response options provide contextual cues that influence participants’ interpretations of the stimuli. They also speculated that the RMET performance differential typically observed between autistic and non-autistic individuals may reflect difficulties in concept learning (i.e. the ability to categorize objects based on common attributes) rather than perspective-taking difficulties. For example, it is possible that non-autistic individuals are more likely to use deductive reasoning to select the correct answer (e.g. “It looks like an unpleasant emotion, so the answer can’t be ‘happy’”). As there is some suggestion that autistic individuals may have difficulty with category learning and generalization of concepts (e.g. Klinger & Dawson, 2001), autistic individuals may rely less on such compensatory strategies to complete the multiple-choice RMET compared to non-autistic individuals, thus resulting in lower scores. Such findings raise concerns about the construct validity of the multiple-choice RMET as a theory of mind measure.

Betz et al. (2019) argued that inferences drawn from prior research using the RMET be re-evaluated. One such inference is that autistic adults perform more poorly on the RMET than non-autistic adults due to difficulties with theory of mind that are considered to characterize autistic individuals (Baron-Cohen, Wheelwright, Skinner, et al., 2001). Yet, recent research suggests that difficulties with theory of mind are not universal among autistic adults (Brewer et al., 2017; Gernsbacher & Yergeau, 2019). Given the aforementioned limitations in the construct validity of the multiple-choice RMET, it is possible that these group differences reflect differences in verbal ability or concept learning, rather than theory of mind. It is thus important for accurate measures of theory of mind to be developed, as such tools would enable clinicians to better understand the specific needs of their clients and the potential factors that may be contributing to their difficulties with social communication and interaction.

We (1) replicated Betz et al.’s (2019) examination of response format on RMET performance, but used both autistic and non-autistic adult samples, (2) compared the discriminant validity of the multiple-choice and free-report RMET for autistic and non-autistic adults, and (3) examined the convergent validity of both RMET formats using an independent theory of mind measure, the Adult Theory of Mind test (A-ToM-Q; Brewer et al., 2022).

## Method

### Participants

As both Cassels and Birch (2014) and Betz et al. (2019) reported large effect sizes of response format on RMET performance, we targeted a sample size of 128 participants to detect a medium effect size (*f* = .25) at alpha = .05 and power = .80 (G*Power 3.1; Faul et al., 2007). One hundred and ninety-five participants from Australia, Canada, New Zealand, United Kingdom, and United States were recruited using the online crowdsourcing platform, Mechanical Turk (MTurk). Thirteen respondents were deleted due to suspected use of an automated system (nonsensical or grossly irrelevant text entered in text boxes). Of those remaining, 91 reported a formal diagnosis of autism from a trained professional and specified the type of professional who made the diagnosis (e.g. psychologist, pediatrician) and their age at diagnosis. To validate these diagnoses, participants’ Autism Spectrum Quotient (AQ; Baron-Cohen, Wheelwright, Skinner, et al., 2001) scores were considered. Twenty-one of the 91 participants reported a diagnosis of autism but did not score above the clinical cut-off of 26 (Kurita et al., 2005; Woodbury-Smith et al., 2005) on the AQ (*M* = 20.90, *SD* = 3.22); their data were excluded from analyses. Twenty participants who scored 26 or higher on the AQ (*M* = 35.45, *SD* = 7.13) but did not confirm an autism diagnosis were also excluded. The final sample comprised 70 autistic (32 male, 37 female, one non-binary) and 71 non-autistic participants (46 male, 24 female, one non-binary). All participants were fluent in English, with 139 participants indicating that English was their first language. No information was collected on participants’ ethnicity and socioeconomic status.

### Materials

#### Ten-item Reading the Mind in the Eyes Test (RMET)

The 10-item RMET (Olderbak et al., 2015) presents respondents with 10 images of a pair of human eyes and asks them to judge the emotion captured in the image. The 10-item version of the RMET was used as it demonstrates better unidimensionality and internal consistency than the original 36-item version (Olderbak et al., 2015). The multiple-choice RMET had four response options per item, accompanied by a glossary defining those options. In the free-report format, participants typed their answer in a text box (participants in the free-report condition were not provided with a glossary). Free-report responses were scored by three independent raters against the Merriam-Webster online thesaurus and dictionary as meeting either a stringent, lax, or boundary definition of the target emotion, or as not meeting the definition. For example, on Item 3 (Skeptical), “confused” was considered a boundary definition, “leery” a lax definition, and “suspicious” a stringent definition. (The complete scoring sheet can be accessed at https://osf.io/93sjm/). On all but one response, at least two of the three raters provided the same score. Disagreements were discussed until consensus. Responses meeting a stringent or lax definition were scored correct; all other responses were scored incorrect. RMET scores range from 0 to 10; higher scores indicate higher levels of theory of mind.

#### Autism Spectrum Quotient (AQ)

The AQ (Baron-Cohen, Wheelwright, Skinner, et al., 2001) is a 50-item self-report measure of autistic traits. Scores range from 0 to 50; higher scores indicate a higher degree of autistic traits. A cut-off score of 26 has been found to have good sensitivity and specificity in discriminating autistic and non-autistic individuals (Kurita et al., 2005; Woodbury-Smith et al., 2005).

#### Adult Theory of Mind–Quick (A-ToM-Q)

The social subscale of the Adult Theory of Mind–Quick (A-ToM-Q) test (Brewer et al., 2022) requires respondents to view six videos of interpersonal interactions, each followed by a multiple-choice question (four alternatives) probing their interpretation of subtle social nuances (e.g. *faux pas*, sarcasm, white lie). Scores on this subscale range from 0 to 6; higher scores indicate greater theory of mind. The A-ToM-Q’s social subscale correlates significantly with other theory of mind and criterion-related measures (Brewer et al., 2022). Divergent validity is indicated by the absence of correlations with measures that differentiate autistic and non-autistic samples but do not demand perspective taking, such as the Mini-SPIN (Brewer et al., 2022). Discriminant validity of the A-ToM-Q is evidenced by autistic adults being more strongly differentiated from non-autistic adults on the social than its physical (or control) subscale (Brewer et al., 2022).

#### Self-Administered Vocabulary IQ Test (SA-VIQT)

The SA-VIQT is an online verbal IQ test from the Open-Source Psychometrics Project. On each of 45 items, participants are presented with five words and select the two that mean the same. Correct responses receive one point, while incorrect responses are deducted one point. “Don’t know” responses are neither awarded nor deducted points. The SA-VIQT provides an overall verbal IQ (VIQ) score ranging from 40 to 160. It is moderately correlated with the Wechsler Abbreviated Scale of Intelligence (WASI-II) (Wechsler, 2011), Verbal Comprehension Index (VCI; *r* = 0.48), WASI-II FSIQ-2 (*r* = 0.54), and WASI FSIQ-4 (*r* = 0.53), suggesting the SA-VIQT’s viability as a quick research screening measure of verbal IQ (Logos et al., 2021).

### Design

RMET performance was examined using a 2 (Group: autistic, non-autistic) × 2 (Response Format: multiple-choice, free-report) between-subjects design.

### Procedure

This project was approved by the Flinders University Human Research Ethics Committee; participants read a study information sheet and gave informed consent. The study was administered using Qualtrics. Participants provided demographic information and indicated if they had received a formal diagnosis of autism. Two attention checks were used to identify the use of robots or automated systems. Participants completed the AQ and A-ToM-Q social subscale, were randomly allocated to either the free-report or multiple-choice RMET, and then completed the SA-VIQT. Participants received an honorarium as compensation for their time.

### Community involvement statement

Two of the authors are practicing clinical psychologists who consult with autistic adults and children.

## Results

As shown in Table 1, the autistic group scored higher on the AQ and lower on the A-ToM-Q than the non-autistic group. There was no significant group difference in VIQ, but the non-autistic group was significantly older than the autistic group. The correlations between all variables are provided in Supplementary Materials (p. 2).

**Table 1.** Descriptive statistics for age, AQ, VIQ, and A-ToM-Q for the two groups.

Measure	Autistic (*n* = 70)	Non-autistic (*n* = 71)	*p*	*d*
Age in years (*M*, S*D*)	32.23 (8.38)	39.87 (12.56)	<0.001	–0.72
AQ (*M*, *SD*)	36.64 (7.12)	16.65 (5.80)	<0.001	3.08
VIQ (*M*, *SD*)	98.12 (22.09)	105.07 (17.83)	0.17	–0.35
A-ToM-Q (*M*, *SD*)	3.03 (1.82)	4.93 (1.25)	<0.001	–1.22

*SD*: standard deviation; AQ: Autism Spectrum Quotient; VIQ: Vocabulary IQ; A-ToM-Q: Adult Theory of Mind–Quick.

*p*-values derived from *t*-tests with Bonferroni correction. VIQ scores absent for two autistic participants.

A 2 (Group: autistic, non-autistic) × 2 (Response Format: multiple-choice, free-report) between-subjects analysis of variance (ANOVA) revealed a main effect of response format on RMET scores, with higher scores on the multiple-choice than the free-report version, *F*(1, 135) = 294.78, *p* < 0.001, η_p_^2^ = .69. The autistic group scored lower than the non-autistic group on both the multiple-choice and free-report RMET, *F*(1, 135) = 37.35, *p* < 0.001, η_p_^2^ = .22 (see Table 2). A significant Group × Response Format interaction, *F*(1, 135) = 4.17, *p* = 0.04, η_p_^2^ = .03, reflected larger differences between the autistic and non-autistic groups on the multiple-choice than free-report RMET.

**Table 2.** Mean (standard deviation) and median Reading the Mind in the Eyes Test (RMET) scores by response format and group.

Response format	Autistic	Non-autistic	Total	*d* [95%CI]
Multiple-choice				
*M* (*SD*)	6.03 (2.42)	8.45 (1.50)	7.31 (2.32)	1.22 [0.74, 1.72]
*Mdn*	5.50	9.00	8.00	
*N*	34	38	72	
Free-report				
*M* (*SD*)	1.54 (1.12)	2.75 (1.72)	2.12 (1.55)	0.84 [0.34, 1.34]
*Mdn*	2.00	3.00	2.00	
*N*	35	32	67	

CI: confidence interval.

Multiple-choice RMET scores were missing for one participant from each group. *d* denotes the effect size of the mean difference between autistic and non-autistic participants on each RMET format. Scores on both response formats of the RMET can range from 0 to 10.

For the overall sample, multiple-choice performance was correlated with verbal IQ, *r*(69) = 0.34, *p* = 0.003. Unsurprisingly, given the non-autistic group performed near ceiling, their multiple-choice RMET performance was not correlated with verbal IQ, *r*(35) = 0.07, *p* = 0.67. For the autistic group, the nonsignificant correlation indicated a moderate effect size, *r*(32) = 0.33, *p* = 0.06.

Free-report RMET performance was also significantly correlated with verbal IQ for the overall sample, *r*(64) = 0.29, *p* = 0.02, although the correlation for the autistic group, which performed near the floor, was negligible, *r*(32) = 0.07, *p* = 0.69. The correlation for the non-autistic group indicated a moderate effect size, *r*(30) = 0.45, *p* = 0.01.

Given that verbal IQ and age were significantly correlated with RMET performance, analyses were repeated with verbal IQ and age as covariates. The main effects of response format, *F*(1, 131) = 301.81, *p* < 0.001, η_p_^2^ = .70, and group membership, *F*(1, 131) = 28.80, *p* < 0.001, η_p_^2^ = .18, on RMET scores remained. However, the Response Format × Group interaction on RMET scores disappeared, *F*(1, 131) = 2.89, *p* = 0.09, η_p_^2^ = .02.

There was a strong correlation between the multiple-choice RMET and the A-ToM-Q in the overall sample, *r_s_*(70) = 0.59, *p* < 0.001, with verbal IQ controlled, *r_s_*(69) = 0.54, *p* < 0.001. The free-report RMET had a weak-moderate correlation with the A-ToM-Q in the overall sample, *r_s_*(65) = 0.28, *p* = 0.02. Controlling for verbal IQ, the latter correlation was no longer significant, *r_s_*(64) *=* .22, *p* = 0.07, but the coefficient was only slightly, and not significantly (*z* = -0.53, *p* = 0.30), lower. The correlation between free-report RMET and A-ToM-Q was significantly weaker than the correlation between multiple-choice RMET and A-ToM-Q (with verbal IQ controlled), *z* = -2.18, *p* = 0.02; however, with free-report RMET performance relatively close to the floor (regardless of group), this pattern is unsurprising.

## Discussion

Consistent with Cassels and Birch (2014) and Betz et al. (2019), participants performed better on the multiple-choice than the free-report RMET, suggesting that the multiple-choice format enables the use of additional strategies. Regardless of RMET response format, the RMET decisively discriminated autistic and non-autistic adults. Although the difference between groups was larger for the multiple-choice format than the free-report format, this difference was no longer statistically significant with VIQ controlled. Moreover, although VIQ was correlated with both multiple-choice and free-report performance, controlling for VIQ did not undermine the ability of either version to discriminate the two groups.

In addition, examination of the concurrent validity of both RMET formats revealed that multiple-choice performance correlated strongly with the A-ToM-Q. Although free-report performance was not as strongly correlated, this likely reflects free-report performance being close to the floor. These correlations with A-ToM-Q performance remained consistent after controlling for VIQ. In sum, our findings provide evidence for the concurrent validity of both versions and suggest that the validity of the RMET is not dependent on verbal ability. Given the demanding coding requirements for scoring free-report RMET responses, the multiple-choice RMET is the more accessible, efficient, and economical option.

### Limitations

First, we did not obtain evidence that participants had received a formal diagnosis of autism, relying instead on self-reports of a diagnosis and AQ scores. Second, the SA-VIQT, a quick screening measure of VIQ is not as rigorous as a full-scale verbal IQ measure such as the Wechsler scales. Third, although our results provided promising evidence of the RMET’s concurrent validity with the A-ToM-Q, we note that the A-ToM-Q’s stimulus videos depicting social interactions include (inter alia) the target individuals’ facial expressions. Thus, it is possible that cues from the eye region may contribute to a degree of shared variance between RMET and A-ToM-Q scores. One way to examine this possibility would be to isolate or pixelate the eye region of the characters in the A-ToM-Q stimuli.

## Conclusions

Our results indicate that both the multiple-choice and free-report versions of the RMET differentiated autistic and non-autistic adults irrespective of verbal ability. However, given its ease of administration, the multiple-choice format offers clear practical advantages over the free-report format.

## Supplemental Material

sj-docx-1-aut-10.1177_13623613231167226 – Supplemental material for Response format changes the reading the mind in the eyes test performance of autistic and non-autistic adults Click here for additional data file. Supplemental material, sj-docx-1-aut-10.1177_13623613231167226 for Response format changes the reading the mind in the eyes test performance of autistic and non-autistic adults by Alliyza Lim, Neil Brewer, Denise Aistrope and Robyn L Young in Autism
