# Supplementary material for: Response format changes the reading the mind in the eyes test performance of autistic and non-autistic adults
Source: Autism. 2023 May 3;27(8):2560–5. doi: 10.1177/13623613231167226 (PMC10576897; doi:10.1177/13623613231167226)
Supplement: sj-docx-1-aut-10.1177_13623613231167226 – Supplemental material for Response format changes the reading the mind in the eyes test performance of autistic and non-autistic adults [file sj-docx-1-aut-10.1177_13623613231167226.docx]

**Figure S1**

*Flow Diagram of Participant Inclusion and Exclusion Criteria*

**
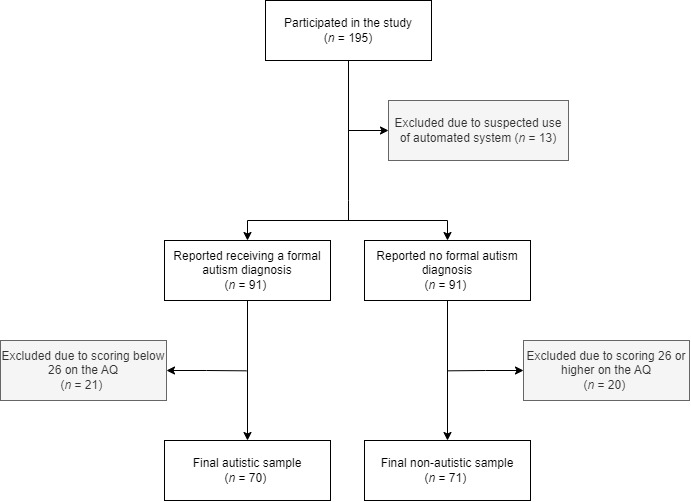
**

**Table S1**

*Correlations of RMET Score, Age, Verbal IQ, and AQ Score by RMET Response Format and Group Membership*

|  |  |  | RMET | Age | VIQ | AQ |
| --- | --- | --- | --- | --- | --- | --- |
| Multiple-Choice Format | Combined Sample (*n* = 74) | RMET | - | .11 | .34** | -.48*** |
|  |  | Age | .11 | - | .411*** | -.33** |
|  |  | VIQ | .34** | .411*** | - | -.17 |
|  |  | AQ | -.48*** | -.33** | -.17 | - |
|  | Autistic Sample (*n* = 35) | RMET | - | .21 | .33 | -.10 |
|  |  | Age | -.21 | - | .23 | .07 |
|  |  | VIQ | .33 | .23 | - | .37* |
|  |  | AQ | -.10 | .07 | .37* | - |
|  | Non-Autistic Sample (*n* = 39) | RMET | - | .02 | .07 | -.02 |
|  |  | Age | .02 | - | .53*** | -.16 |
|  |  | VIQ | .07 | .53*** | - | -.30 |
|  |  | AQ | -.02 | -.16 | -.30 | - |
| Free-Report Format | Combined Sample (*n* = 67) | RMET | - | .35** | .29* | -.36** |
|  |  | Age | .35** | - | .26* | -.27* |
|  |  | VIQ | .29- | .26* | - | .06 |
|  |  | AQ | -.36** | -.27* | .06 | - |
|  | Autistic Sample (*n* = 35) | RMET | - | .05 | .07 | .06 |
|  |  | Age | .05 | - | .15 | .21 |
|  |  | VIQ | .07 | .15 | - | .50** |
|  |  | AQ | .06 | .21 | .50** | - |
|  | Non-Autistic Sample (n = 32) | RMET | - | .36* | .45** | -.21 |
|  |  | Age | .36* | - | .34 | -.21 |
|  |  | VIQ | .45** | .34 | - | -.25 |
|  |  | AQ | -.21 | -.21 | -.25 | - |

*Note*. Multiple-choice RMET scores were missing for one participant from each group. VIQ score was missing for one autistic participant in the free-report condition and one non-autistic participant in the multiple-choice condition.

* *p* ≤ .05, ** *p* ≤ .01, *** *p* ≤ .001 -
